# Workflow disruptions in robot-assisted surgery

**DOI:** 10.1007/s11701-023-01728-2

**Published:** 2023-10-10

**Authors:** Shing Wai Wong, Philip Crowe

**Affiliations:** 1 Department of General Surgery, Prince of Wales Hospital, Sydney, NSW Australia; 2 Randwick Campus, School of Clinical Medicine, The University of New South Wales, Sydney, NSW Australia

**Keywords:** Workflow, flow disruptions, robotic surgery, situation awareness

## Abstract

Surgical flow disruptions are unexpected deviations from the natural progression which can potentially compromise the safety of the operation. Separation of the surgeon from the patient and team members is the main contributor for flow disruptions (FDs) in robot-assisted surgery (RAS). FDs have been categorised as communication, coordination, surgeon task considerations, training, equipment/ technology, external factors, instrument changes, and environmental factors. There may be an association between FDs and task error rate. Intervention to counter FDs include training, operating room adjustments, checklists, teamwork, communication improvement, ergonomics, technology, guidelines, workflow optimisation, and team briefing. Future studies should focus on identifying the significant disruptive FDs and the impact of interventions on surgical flow during RAS.

## Introduction

The introduction of robotic-assisted surgery (RAS) has posed new challenges for the surgeon to develop new psychomotor and hand–eye coordination skills as well as for the team to adapt communication, teamwork, and coordination skills [1, 2]. Despite improvements of visualisation, manipulation and posture for the surgeon, the surgical team needs to adjust to the separation of the surgeon from the patient and team members [3, 4]. Separation of the surgeon may reduce situation awareness but immersion in the binocular three-dimensional view may help reduce distractibility [3]. Team members need to adjust to the changed operating room layout related to the housing of the large robot components, which can result in obstructions, disorganisation, and unnecessary movement [5].

Human factors research pertaining to RAS is the study of the relationship between people and the robotic system [1, 6]. Human factors research postulates that a combination of environmental factors and not surgeon or patient factors alone contribute to errors [6]. Accumulation of minor events can reduce the compensatory mechanisms of the surgical team to cope with a major event and thus predisposing them to errors [7]. Weigmann et al. found teamwork and communication flow disruptions (which accounted for 52% of all events) as the most reliable predictor of error [7].

Workflow can be defined as the organisation of activities that enables the provision of surgery [8]. Most RAS research focussed on patient outcomes, ergonomics, and surgeon technical skills. Research in factors which affect surgical flow and social/cognitive skills may be more important in influencing safety concerns of RAS. Introduction of modern technology has consequences not only for the surgeon but also for the other team members because of the complex changes in division of labour [3].

Flow research theory is connected to mindfulness or being fully attentive to the task, self-control with merging of action and awareness, where individual performance is seen at its peak [9]. Natural flow occurs during surgery when the procedure progresses with ease and fluidity. Surgical flow disruptions are unexpected deviations from the natural progression which can potentially compromise the safety of the operation. Continuous distractions or persistent stressors (such as background noise, time pressure or fatigue) are not usually classified as flow disruptions (FDs) [10].

FDs have been categorised as communication, coordination, surgeon task considerations (such as leadership and decision making), training, equipment/ technology, external factors (such as situational awareness), instrument changes, and environmental factors (such as spatial configuration) [11–13]. FDs can be assessed by quantitative counts and/or by qualitative free-text notes [14]. The advantages of qualitative data are more detailed analysis of specific events (which can be reviewed later) and to ensure no false positives with the quantitative counts.

Studies have revealed 20% of operating time were attributed to FDs, 14% of interruptions were potentially avoidable, each FD added 2.4 min to the operation time, and 30% of all FDs were high impact [1, 10, 15, 16]. Severity of FDs have been classified by how many team members were affected by the event and whether the distraction needed attention [16]. FDs may affect mental workload, performance, and teamwork. However, not all disruptions are significant or avoidable. Most FDs were initiated by team members who usually avoided them when absolute concentration was required [12]. Some disruptions are potentially beneficial in promoting team relaxation, education, or surgeon decision making.

A review article found a significant gap between issues and solutions in RAS [5]. Most articles suggested an intervention based on study results and some discussed the development, implementation, and evaluation of an intervention. The categories of intervention included training, adjustments to the operating room, checklists, teamwork, communication improvement, ergonomics, use of technology, implementation of guidelines, optimisation of workflow, and team briefing.

The causes of FDs are interrelated and often co-exist simultaneously because they are related to the separation of the surgeon. MEDLINE (via PubMED) and Google Scholar were searched. Inclusion criteria were RAS and workflow/flow disruptions/non-technical skills. Other articles were found from relevant references in the retrieved articles. The most frequent FD categories are presented individually but overlap is common because of the direct relationships between the sub-classifications. The impact of FDs and interventions to counter FDs are addressed.

### Teamwork and division of labour

Effective teamwork reduces the impact of FDs. Koch et al. found an experienced robotic surgery team decreased external disruptions during “high-risk” surgical tasks [12]. Allers et al. found that higher team familiarity (based on duration and number of procedures team members worked together) and cohesiveness reduced FDs by better anticipation and preparation [15]. Sexton et al. found anticipation resulted in 8% reduction of operating time and active engagement by a team with high familiarity resulted in fewer inconveniences [17]. Anticipated requests were noted to be five times shorter than non-anticipated requests.

RAS changes the division of labour within surgical teams, with the surgeon doing more and the assistant role less clearly defined [18]. With RAS, the assistant and scrub nurse (who now work as a unit) have an additional task of communicating effectively with the surgeon about the situation at the patient interface. The individual experience, team relationships, and negotiation context may change the division of labour between assistant and scrub nurse. There are hierarchical and boundary blurring influences of division of labour with the surgeon’s physical separation from the team. Role-specific and team training can enhance knowledge, communication, and coordination skills [1, 14, 16].

### Communication

The quality and quantity of information exchanged defines communication. Studies have revealed a significant increase in verbal communication during robotic compared with laparoscopic surgery, related to less nonverbal communication [3]. Surgeon control of the camera and three other robotic arms may result in more independence and reduced need for communication. Erroneous or inefficient communications which cause FDs may be case relevant or irrelevant [19].

Communication FDs have been subclassified into nine categories: repeat, misunderstanding, clarification, unacknowledged, microphone, distraction, discussion, conflict, and noise [20]. The most frequent communication problem was related to need to repeat information, which occurred when the assistant and scrub nurse could not hear clear directions from the surgeon and/or to whom they were directed [19, 21]. Communication between the surgeon and the bedside team is potentially most critical during instrument exchanges.

Lack of eye contact and nonverbal cues inhibit effective communication during RAS [22]. Nonverbal communication such as gestures, eye gaze direction, facial expressions, and body orientation are more ambiguous and less efficient compared with verbal communication [23]. Nonverbal interactions differed significantly by pair: 66% time occurrence for the surgeon-assistant (who have a shared console view), 50% for the assistant-scrub nurse (who can interact face-to-face), and 25% for the surgeon-scrub nurse (because the surgeon has little visual evidence of scrub nurse activities) [23]. Nonverbal communication can occur by instrument movement on screen, camera view change and display indicators on screen. A motivated team of high familiarity, process consistency, and experience can reduce the need for verbal communication because of better anticipation and preparedness for the surgeon requests [22, 24]. The challenge is not only hearing the instructions but interpreting it correctly. Communication is dependent on the available mode and the purpose of the interaction. Communication is, however, not dichotomous but multimodal and relies on multiple verbal and nonverbal interactions embedded in a certain situation to transfer information effectively.

Complete comprehensive requests (made 47% of the time) resulted in a significant decrease in action time [24]. Strategies to overcome challenges of communication include improved clarity, read-back, and loop closure [21, 22]. More specific requests with standardized taxonomy, agreed terms, or scripted speech patterns can result in faster action and lower number of inconveniences [21, 24, 25]. Directional cues can be confusing because of the different physical context and frames of reference used by surgeon and assistant [26]. Use of anatomical or operating room references can reduce misunderstandings. Adaptation of surgical and communication style when operating with new assistants, restriction of case-irrelavant communication, limitations of the number and frequency of visitors are other methods which have been advocated to maintain workflow [7]. The benefits of repeating back instructions include increased situation awareness, reduced anxiety of requests being unheard, and ability to correct misunderstandings [3]. The physical separation relies on bidirectional speakers on the surgeon console and robot cart for communication. Projected muffle sounds from the surgeon and returned staff chatter to the surgeon posed communication challenges [22, 25]. Noise-cancelling headsets have been used to improve voice clarity and reduced ambient noise [27].

### Coordination

Coordination disruption has been defined as any lapse in teamwork to prepare for or conduct surgery that affects surgery flow [11]. This has been defined as failures to have the right people, with the right tools, in the right place, at the right time [20]. Coordination FDs have been subclassified into 12 categories: supplies retrieval, training support, human error, waiting, supplies accommodation, unavailability, troubleshooting, equipment adjustments, robot-specific, patient accommodation, equipment move, and training support [20]. The need for the scout nurse to find equipment located outside the operating theatre was the most common reason for coordination FDs [14]. During the robot docking stage, coordination-related FDs were the most common disruptions that occurred [16].

Mutual support with anticipation of other team members’ needs and the ability to shift workload among members to achieve balance resulted in more efficient coordination [17, 28]. Scrub nurse anticipatory movements without surgeon instruction are compromised when the nurse is physically separated from the surgeon [3]. The physical separation makes it difficult for the theatre team to monitor the surgeon’s actions and physical gestures but information from two-dimensional screens can compensate [21].

Preparation with a briefing, verbal acknowledgements, specificity in language, clear paths for uninhibited movement for staff and equipment, anticipation of potential collisions, assigning look-out roles, and consistency of roles to increase familiarity with specific tasks can enhance coordination. Use of a checklist (to overcome fallibility of human memory and to avoid missing steps) in RAS has been shown to increase preparedness and confidence of team members, improve teamwork and efficiency and decrease workflow interruptions [29]. Better coordination can ensure instrument supply availability, enhanced problem solving and safer manoeuvring of the robot.

### Leadership

Leadership has been defined as a process when the team is directed in a way that makes it more cohesive and coherent in achieving an objective [30]. Other important components of leadership include team performance assessment, task assignment, team knowledge and skill development, motivation, organisation, assertiveness, and positive team atmosphere establishment [28, 31]. The relationship between leadership intervention and leadership improvements in surgery has been demonstrated but this has not necessarily translated to improved patient outcome [32]. Increased RAS surgeon experience has been shown to reduce FD rate especially during the console phase of the surgery [6, 11].

Cofran et al. showed that leading and supporting the rest of the team may be equally important as improving individual technical skills for the surgeon to promote efficiency and safety in RAS [14]. Surgeon leadership involves ensuring team members are comfortable and confident in voicing concerns and staying engaged. Assistants may not speak up because they are often the least experienced or consistent team member [22]. Inexperienced staff often find separation from the surgeon stressful because of loss of nonverbal communication, loss of control, and intermittent blockage of vision screens. Disengagement from lack of active involvement can result in fluctuation of attention and contribute to side-talks.

### Decision-making

Intraoperative decision making involves a continuous cycle of preoperative plan, assessment of the situation with respect to the possibilities of different actions, reconciliation of added information with existing information and later implementation of a revised course of action [3]. Surgeons pausing surgery for decision making and taking time out to think may be disruptive in terms of flow but is also a safety precaution [1, 33].

Collaborative team situation awareness may compensate for the surgeon’s separation. Different members of the team have access to different information. Judicious sharing of this information in a “cognitive system” rather than relying on individual cognition may overcome the difficulties of separation and improve decision making [3]. Surgeon immersion in the console may reduce distractibility. Case-irrelevant communication and interruptions can result in information overload and negative decision-making performance. Stress may impair judgment and decision making. Reduced physical discomfort and improved manoeuvrability of the instruments may reduce stress, but longer operation duration has been associated with mental fatigue and stress. The increased cognitive load from controlling three instruments and the camera may counteract the benefits of less distractions. Lack of tactile feedback associated with RAS may also affect surgeon decision making. However, experience and three-dimensional vision may help overcome this lack of haptic feedback.

Positioning of the console so that the surgeon has a clear view of the patient can enhance surgeon situation awareness and decision making [34]. The open console design of the newer robotic surgical systems facilitated better communication, because the surgeon was closer to the bedside [35].

### Training

Training-related FDs involves instruction by the surgeon and occurs in training cases. They have been classified into procedure-specific, instrument, anatomy, and robotic surgery-specific instructions [20]. A common training FD is related to surgeons instructing assistants where to place ports [6, 11, 19]. In addition, the experienced scrub nurse may need to take on the extra training responsibility of an inexperienced surgical assistant during RAS [18]. Intraoperative education (e.g., regarding anatomy) may be beneficial effect by improving engagement.

### Equipment/technology

Equipment-related FDs include visual problems (from lens fogging), robot arm, unfamiliarity, insufflation problems, equipment arrangement, robot limitations, inadequate equipment, and sterility issues [20]. Malfunctioning or expired instruments were examples of equipment issues and occurred more often with RAS [11]. Another example was obstruction of bedside team member views by the robot arms [26]. Instrument changes are more complicated during RAS compared with laparoscopic surgery and require more anticipation and consideration of the pathway of the instruments, because there is no direct visualisation despite the enabling technology which allows more location precise replacement of an instrument.

### Situation awareness

Situation awareness has been defined as the perception of components in the environment, and the comprehension of their meaning [3]. The tendency for surgeons to immerse themselves in the console and block out the rest of the operating theatre may reduce situation awareness [22, 26, 36]. Console immersion may be more common in the learning phase because of the need for more concentration [36].

The surgeon cannot see the operating table, patient, robot, and team clearly [26]. Robotic arm clashes are common consequences of reduced surgeon situation awareness [34]. Collisions between robotic arm, bedside assistant arm and patient during RAS are common [37]. Previous experience as a bedside assistant can help robotic surgeons appreciate some of the difficulties encountered by the assistant. Mutual performance monitoring is the capacity to develop common understandings of the team environment and apply appropriate strategies to monitor team member performance accurately [28]. Other team members can compensate for reduced surgeon situation awareness which requires trust, effective communication, customs, and practice [18, 34].

The reduction in task load for the bedside team because the surgeon can control more instruments can lead to disengagement [26]. The bedside team can troubleshoot and communicate more promptly if they stay alert and engaged. Learning to use the technology and educational focus were methods which engaged the scrub nurse and assistant to compensate for their lesser roles [21, 22].

### Spatial configuration

Workspace management with improved configuration of the room layout within the fixed space can reduce the potential for collisions and obstructions as well as allow uninhibited movement. The size and layout of the operating room needs to consider the size of the patient cart, surgeon console, vision cart and associated cables [20]. The cluttering of equipment, wires and tubes may hinder movement flow [38]. Ahmad et al. found that 50% of all tracked movements could have been avoided if the operating room setup were optimised to cater toward team member tasks [38]. The purposes for avoidable pathways were primarily related to delivery of equipment by the scout nurse to the scrub nurse. Improvements in room layout repositioning included better access to supply zones, targeting of unobstructed views, and optimisation of monitor locations. The storage of supplies should also consider the frequency and sequence of equipment use. Wireless transmission, better accessibility to bins, development of an integrated operating table and use of sliding doors were other considerations which can improve movement efficiency [38].

### Resilience

Resilience has been defined as an adaptive response to unexpected events while maintaining reliable performance [12]. Support for resilience has been attributed to surgeon behaviour but did involve other factors [39]. Person-related resilience supports included provision of skills training (e.g., advising caution), personality (e.g., calmness), anticipatory planning, effective communication, and strong leadership (e.g., positive feedback). Task-related resilience support included optimising ergonomics. Tools and technology resilience supports were related to availability, usability, and effective functionality. Advantageous organisational resilience supports included a strong safety culture, adaptation of team responsibilities, and commitment to intra-operative education. Important environmental supports considered include spatial requirements, setup, walking paths and ambient conditions [40].

## Impact of workflow disruptions

Three distinct categories of surgical outcomes because of FDs have been studied: surgical process, team, and patient [10]. Process outcomes studied were operating time, errors, performance metrics and costs. Team outcomes included mental workload, teamwork, communication, non-technical skills, stress, and perceived distraction. Patient outcomes included complications, and surgical site infections. Studies have shown an association between FDs, task error rate, unsafe decisions, mental workload, and operating time [19]. There may be bidirectional influences with extended procedure time resulting in increased occurrence of FDs. High workload (the cost incurred to achieve a particular level of performance) can result in higher stress levels which can affect performance. Addressing FDs can lead to a more efficient process with reduced operating time, better teamwork and job satisfaction, and reduced complication rates [14].

The relationship between FDs and outcomes may not be direct and may not affect the patient [14]. Koch et al. reported no conclusive evidence that FDs had a harmful impact on patient outcomes possibly because of team resilience [12]. Some FDs, such as immersion and taking time out to think, may have a beneficial effect. Immersion in the surgeon console may reduce surgeon awareness of disruptions and distractions which can lead to information overload. In addition, some FDs were unavoidable or essential, e.g., relaying of urgent information.

## Conclusion

The ergonomic advantages of RAS for the surgeon may be offset by teamwork disadvantages related to the separation of the surgeon from the team. There are trade-offs between efficiency, safety, and training. Most studies focussed on disruptions rather than maintenance of workflow. Future studies should focus on identifying the significant disruptive FDs and the impact of interventions on surgical flow during RAS.

## Data Availability

Not applicable. Narrative review article.
